# Antimicrobial use in sows in Finland and its association with herd characteristics

**DOI:** 10.1186/s40813-025-00447-4

**Published:** 2025-07-14

**Authors:** Kristina Ahlqvist, Camilla Munsterhjelm, Anna-Riia Holmström, Minna Kujala-Wirth, Vera Talvitie, Anna Valros, Mari Heinonen

**Affiliations:** 1 Research Centre for Animal Welfare, Department of Production Animal Medicine, Faculty of Veterinary Medicine, University of Helsinki, Helsinki, 00014 Finland; 2 Faculty of Pharmacy, Division of Pharmacology and Pharmacotherapy, Faculty of Veterinary Medicine, Department of Clinical Equine and Small Animal Medicine, Helsinki, 00790 Finland; 3 Animal Health ETT, Seinäjoki, 60101 Finland

**Keywords:** Antibiotics use, Biosecurity, Sows, Pigs, mg/PCU, Indication, Risk factors

## Abstract

**Background:**

Antimicrobial use (AMU) in food-producing animals affects development of antimicrobial resistance. Previous studies have shown that AMU for pigs varies considerably between herds and countries. Finland has relatively low AMU in pigs, although pigs are the main species treated with antimicrobials. In Finland, the use of medicines for pigs is recorded in the national web-based herd health and welfare register Sikava. We aimed to qualitatively and quantitatively describe AMU in Finnish sows using anonymous herd health data and to identify indications for antimicrobial treatment, antimicrobial agents used for each indication, and farm-level risk factors associated with AMU. Forty-eight randomly selected herds with more than 100 sows were selected from the herd register of 905 herds. The register data included AMU in sows, biosecurity evaluations, welfare index calculated by the Sikava system, and scores given by veterinarians during veterinary health care visits in 2022. Visiting veterinarians collect information on housing and environmental conditions, animal health, and welfare using a standardized protocol and record their findings electronically in the herd health register. Farmers record AMU in sows electronically in the register. Data for this study included the product name, active substance, treatment indication, duration of therapy, number of sows treated, and dosage. AMU in sows was quantified at the herd level as milligrams of antimicrobials administered per population-corrected unit (mg/PCU). Additionally, potential farm-level risk factors were identified.

**Results:**

The median total AMU for the sows was 21.9 mg/PCU (range: 0.3-178.5). The most used antimicrobial was penicillin, and sows were most commonly treated parenterally for locomotory (34% of the treatments), udder (20%), reproductive (12%), and skin (11%) disorders. AMU was higher in large herds than in smaller ones. Piglet producers used more antimicrobials than farrow-to-finish herds, and AMU increased with higher internal biosecurity scores.

**Conclusions:**

AMU in Finnish sows varied widely between herds. Injectable penicillin was the most commonly used antimicrobial, and sows were most frequently treated for locomotory, udder, and reproductive disorders. Large herds, piglet producers, and herds with higher internal biosecurity scores had the highest AMU.

## Background

Antimicrobial resistance (AMR) is one of the greatest threats to global health [1, 2]. The emergence of AMR is partly due to irrational and inappropriate antimicrobial use (AMU) in food-producing animals [2–5]. This irrational use may be due to a lack of knowledge in farmers, inadequate practices from veterinarians, or to mask poor husbandry and biosecurity [6]. This study analyzes both qualitative and quantitative aspects of AMU in sows. Qualitative AMU refers to the descriptive aspects of antimicrobial use, such as the types of antimicrobials used and the reasons for their use. Quantitative AMU involves measuring and analyzing antimicrobial use numerically, including metrics such as the quantity of antimicrobials used (mg/PCU). In Finland, AMU is low in production animals compared with other European countries [7]. However, pigs have the highest AMU in Europe [7] and the second highest in Finland after cattle [7].

Previous studies have shown that AMU for pigs varies considerably between herds [8–13]. Previous Finnish studies have reported the AMU of different age groups separately and found controversial results. In the study of Sali et al., sows had the highest mg/PCU [12], whereas in the study of Yun et al., sows were the age group with the lowest treatment incidence (TI) [13]. The differences between the studies of Sali et al. and Yun et al. may be due to different data collection methods, location, and season. In Finland, the use and record keeping of medicines on pig farms is regulated by legislation (Decrees of the Ministry of Agriculture and Forestry 2008/14/2014 and 2163/14/2014). Most pig producers record medicine use in the national web-based herd health and welfare register Sikava, founded by slaughterhouse companies [14]. Sikava is an ISO-9001-certified health register with the status of a National Quality system approved by the Finnish Food Safety Authority. The register is relatively comprehensive, as about 90% of Finnish pig herds have joined Sikava, representing 97% of the total pork production in Finland [14]. However, no regular national summaries of medicine use are published for different age groups of pigs.

Dunlop et al. reported that mastitis-metritis-agalactia syndrome and off-feed were the most common reasons for antimicrobial treatment of sows in Canada [15]. A Danish study by Jensen et al. showed that the most common treatment indications in sows from 2002 to 2008 were disorders in the locomotory or central nervous system (CNS) and skin and urogenital tract infections, and the most used antimicrobial was penicillin [16]. Another Danish study by Nielsen et al. showed that between 2016 and 2018, most antimicrobial treatments were prescribed for leg, CNS, and skin conditions in sows and suckling piglets [17]. AMU for Finnish production animals is usually individualized. In 2022, 74% of antimicrobials sold for all production animals were used as individual treatments [18]. Sali et al. showed that injectable penicillin was the most used antimicrobial for sows in Finland in 2018 [12]. However, very little has been published on treatment indications and the selection of different antimicrobials for different indications in sows.

Several management practices in intensive livestock farming have been suggested as risk factors for higher AMU and thus for emergence of AMR [19]. Albernaz-Gonçalves et al. defined intensive pig production as raising as many animals as possible in small spaces with short production cycles [19]. Commonly occurring features of intensive housing systems, such as low space allowance, which restrict the expression of natural behavior, cause chronic stress and may increase susceptibility to disease [19]. Previous studies have shown that improvements in housing conditions [5, 19, 20] and farm biosecurity [20–23] can improve animal health and thus reduce the need for antimicrobials.

It is important to identify farm-level factors associated with AMU to find ways to reduce the need for AMU. Our first aim was to qualitatively and quantitatively describe the AMU in sows on Finnish sow farms using anonymous herd health register data. Our second aim was to identify indications for antimicrobial treatments of sows and the different antimicrobial agents used for each indication. Our third aim was to use register data to identify farm-level risk factors possibly associated with AMU.

## Methods

This was a cross-sectional register-based study. All data were collected from the Sikava herd health register for the year 2022 and anonymized.

### Sample size and herd selection

For the sample size calculation, we estimated that 45% of the sows would be medicated. The population size was 172 sow herds with more than 100 sows. We used Epitools Epidemiological Calculator [24] for the calculation. We needed 49 herds to estimate the prevalence of medicated sows (desired precision of estimate 0.12 and confidence level 0.95).

Herds were selected based on herd size, defined as the average number of sows for the previous 12 months. The Sikava register receives monthly numbers of sows present in each herd from the Finnish Swine Registry system authorized by the Finnish Food Authority. Farmers must report monthly numbers of animals, including live and dead (euthanized and found dead) sows, to the system. A sow is defined as a female pig aged ≥ 8 months. According to the study inclusion criteria, all herds with at least 100 sows between July 2021 and July 2022 (186 herds) were selected from the herd register of 905 herds (including all herds in the register). Altogether, 16 herds were excluded due to having animals of several owners in one geographical location, and 12 herds due to missing some medication data. The remaining herds (*n* = 158) were assigned a random number. Based on sample size calculations, the first 50 herds with the smallest random number were selected for the study. During data processing, two additional herds were found to have missing medication data and were therefore excluded, leaving 48 herds for the study: 23 piglet-producing herds, 21 farrow-to-finish herds, and four sow pools [25]. Unfortunately, the final data received from the register included two herds with less than 100 sows (76 and 91 sows), but we were not able to replace these herds with new ones and decided to keep them in the study.

### Collection of register data

Herds registered in Sikava have a healthcare agreement with a herd attending veterinarian responsible for planning, advising, and following the medicine used in the herd. The veterinarian updates the herd health plan at least once a year and includes medication instructions for the commonly seen indications that a farmer can treat. In Finland, veterinarians are allowed to sell medicines to farms without profit, and the farmers make the treatment decision and medicate their animals according to the veterinarian’s instructions. Farmers also keep their medical records in electronic form in the Sikava register.

A farm maintains an agreement with a herd attending veterinarian to conduct regular healthcare visits; however, the veterinarian can delegate other veterinarians to visit the farm as needed and the attending veterinarian may not be the one performing the most frequent visits. For each farm, both the attending veterinarian (*n* = 36) and the veterinarian who had performed most of the herd visits during the study period (*n* = 35) were noted. The total number of veterinarians performing farm visits was 62. According to Finnish legislation (Decree of the Ministry of Agriculture and Forestry 2008/14/2014), veterinarians made herd healthcare visits 4 to 26 times yearly, depending on the herd size. A veterinarian needs to make a herd health visit four, six, eight, twelve, or twenty-six times a year if the number of sows is < 75, 75–299, 300–599, 600–1499, or *≥* 1500, respectively. In the herds belonging to the Sikava system, the veterinarian performs a herd investigation and collects information on husbandry, environment, and animal welfare using a standardized Sikava protocol.

### Housing and environmental conditions

During each herd health visit, the veterinarian assessed air quality, cleanliness of pens, operational condition of housing structures and water and feeding equipment, animal density, amount of bedding and enrichment for lactating and pregnant sows, and amount of nesting material for farrowing sows [14]. According to the protocol, veterinarians scored different housing and environmental conditions as 1 (good), 2 (satisfactory), or 3 (poor). The amount of bedding was scored as good if all animals in the group can lie down in a sleeping area that is clean and dry and the floor is not visible (some bedding material is provided) [14]. The amount of enrichment was scored as good if it was sufficient, appropriate, and safe for the sows, and the sows also actively explored or rooted the enrichment provided [14]. Assessments were made separately for lactating, pregnant, and weaned sows. As scoring was categorical (thus not allowing use of means) and scores varied between visits and within the farm, we used the poorest farm-specific scores given by the herd veterinarian during the follow-up time for statistical calculations of the housing and environmental conditions and combined the scores into two new indices. Firstly, the environment index (score range 5–15) summed the poorest scores for air quality, pen cleanliness, operational condition of housing structures and water and feeding equipment, and animal density. Secondly, the enrichment index (score range 3–9) summed the poorest scores for the amount of bedding and enrichment for lactating and pregnant sows and nesting material for farrowing sows. For both indices, the highest score represented the poorest outcome, and the lowest score represented the best outcome from the perspective of animal welfare.

During each regular herd health visit, the veterinarian assessed the presence of free lactation (including both temporary crating and free farrowing systems) on a scale of yes, no, or not applicable to the farm. This information was scored as a separate factor for statistical analysis, where 0 = no and 1 = yes. The register data did not include information for two herds.

### Body condition score of sows and their shoulder lesions

During each regular herd health visit, the veterinarian visually inspected 30 newly weaned sows (or the entire group if the group size was smaller) and graded their shoulders as having no, minor, or severe lesions. A minor lesion was defined as a visible skin lesion with intact skin, while a severe lesion involved broken skin. Based on these records, we summed the percentage of sows with minor and severe lesions for our study, and we calculated the average of the herd veterinarians’ records during the follow-up time.

The veterinarian also assessed the body condition score (emaciated to thin 1–2, fit 3, fat to very fat 4–5) [26] of 30 pregnant and 10 lactating sows (or the whole group if the group size was smaller). We used thin sows as an explanatory variable in our analysis by calculating the percentage of lactating and pregnant sows with a condition score of 1–2 and determining the average of the herd veterinarians’ records for our study.

### Sow mortality and slaughter rate

Sow mortality was calculated as the percentage of sows reported in the register by the herd owner to have been euthanized or died from the average number of the total sow population during the study year. Slaughterhouses transferred the number of slaughtered sows into the Sikava register, and we calculated the slaughter rate as the percentage of sows slaughtered from the average number of sows in the herd during the study year.

### Sikava welfare index and biosecurity

The Sikava register calculates a Sikava Welfare Index (SWI) based on mostly animal-based indicators, which were selected by applying principles from the Welfare Quality^®^ system for pigs [27]. Sikava register uses the following parameters to calculate the SWI (score range 0-112 points) for sows: body condition score, shoulder lesions, abscesses, housing conditions, vulva biting, amount of bedding, amount of enrichment, mortality, free farrowing, and observations of sow behavior. Traffic light colors describe sow welfare as red if the index is less than 73.47 points (= average index minus two standard deviations, SD), yellow if the index is between 73.47 and 81.23 points (= average index minus one SD), and green if it is more or equal to 81.23 points [14].

A veterinarian conducts annual evaluations of herd biosecurity using the risk-based tool Biocheck.UGent™ (www.biocheck.ugent.be), which has been integrated into the Sikava system. This study included internal, external, and total biosecurity scores (%) in the analyses. Two herds had undergone two biosecurity evaluations during the study year, and the average of their assessments was used in the analyses.

### AMU calculations

AMU data included all sow medications in the study herds during 2022 and included the following information: date, product name, active substance, treatment indication, therapy duration (in days), number of sows treated, and dosage.

AMU in sows was quantified at the herd level as milligrams of antimicrobials administered per population corrected unit (mg/PCU). All AMU was analyzed according to the active substance used in different products: penicillin, amoxicillin, sulfa-trimethoprim, tetracycline, tulathromycin, and lincomycin. Sulfa and trimethoprim were considered as one combined substance because only products containing sulfadoxine plus trimethoprim had been used in the study herds. The use of fluoroquinolones and third- and fourth-generation cephalosporins is restricted by Finnish national legislation [28], and Sikava has also banned these antimicrobials in 2021 [14]. To include all active ingredients in the same figure, we created a log-transformed scale to describe the AMU [29].

According to an EMA report [30], PCU was used to quantify total AMU in sows at the herd level. PCU is a technical unit that estimates the average weight of animals at the time of treatment [30]. The PCU was calculated by multiplying the number of sows by a standardized weight (220 kg) [31]. The use of all antimicrobials per herd was quantified as milligrams based on the concentration and the administered dose.

### Treatment records

We included sow treatments for which the farmer recorded AMU. When two different indications were recorded for the same treatment, the first one used was chosen. The indication codes of Sikava were combined for the study according to Table 1. We defined a treatment course as antimicrobial administration to one sow for one indication during consecutive days.

**Table 1:** Indication codes from Sikava [14] were combined into indication groups for data analysis

Indication group	Sikava register indication codes used
Udder	Disease of mammary gland, milk fever, MMA (mastitis, metritis, agalactia), PDS (postpartum dysgalactia syndrome), agalactia, mastitis, trauma or defect of the udder, poor milk producer
Reproductive	Reproductive and urinary diseases, other reproductive failure, abortion, mummification, metritis or vaginitis (or both), farrowing-related treatment, farrowing induction, farrowing aid, uterine inertia, trauma in genitals, painful farrowing/aggressive sow, vaginal/uterine prolapse, urinary infection, uterine inertia
Locomotory	Locomotory disease, arthritis, other reason for lameness (trauma or infection), trauma in claw(s), claw infection, leg weakness, osteochondrosis
Skin	Skin disease/skin disorder, skin infection, skin trauma, shoulder sore
General infection	Septicemia, erysipelas, meningitis
Digestive	Disease in digestive system, gastric disease, gastric ulcer, rectum prolapse, feeding-related disease, diarrhea
Respiratory	Respiratory disease, other respiratory infection
Fever	Fever without other symptoms, hypothermia
Loss of appetite	Loss of appetite without other symptoms
Other	Tail biting, abscess, accident, injury, other diagnosis, other codes

### Statistical analyses

Statistical analyses were performed in SPSS (IBM SPSS version 29.0, Armonk, New York, USA). Statistical significance was set at an alpha level < 0.05 and a trend at a level < 0.1. The Generalized Linear Mixed Models package (GLMM) was used to investigate the effects of possible collected predictors on AMU. The effect of the veterinarian was accounted for by including a random intercept in the models. As more than one veterinarian typically visited a herd over the study period, two different variables were tested separately to find the one explaining the most significant part of the variability: firstly, the veterinarian who performed the largest number of visits, and secondly, the attending veterinarian. The latter was considered potentially influential as this veterinarian is responsible for medications on the herd and has developed the original medication strategy.

Herd size was categorized, aiming for sufficiently large and approximately equally sized categories, and was divided into small (< 150 sows), medium (> 150 to ≤ 400 sows), and large (> 400 sows) herds. Lists of eligible predictors for GLMM analyses were prepared based on a causal diagram (not shown), including biologically plausible factors that showed at least a tendency to an association with the outcome in univariate tests (Spearman rank correlation ρ).

The three predictors with the highest correlations to mg/PCU (herd size, sows slaughter rate, and Biocheck internal) also correlated with each other to a degree, causing significant collinearity. Separate models were built around each of these three variables. Predictors were added to the models according to a forward stepwise strategy. The process was complicated by relatively strong correlations between many variables (see results section), raising further concerns for collinearity. The Breusch-Pagan test was applied to ensure that collinearity was absent [32]. As more than one alternative significant model could be built, model fit determined the final choice. The final model was approved upon determining normality and homoscedasticity of residuals. Effects of significant categorical variables were investigated further by pair-wise inter-category tests of predicted values, applying a least significant difference correction for multiple comparisons.

## Results

### Herd characteristics

Table 2 summarizes the descriptive statistics for herd type, size, sow mortality, slaughter rates, Sikava welfare index scores, herd biosecurity scores, veterinary assessment of housing and environmental conditions, and animal-based measures from 48 herds. The veterinarian had not evaluated the biosecurity in two herds during the study year.

**Table 2:** Descriptive statistics of herd characteristics and health metrics for 48 study herds investigating antimicrobial use for sows in Finland

Herd type (number of herds)	Piglet-producing herds (23)	Farrow-to-finish herds (21)	Sow pools^1^ (4)	Total (48)
**Herd size, number of sows**				
Mean (SD^2^)	484 (509)	243 (185)	721 (829)	389 (444)
Median	237	175	393	212
Min - Max	76–2068	91–709	147–1953	76–2068
**Sow mortality**,** %**				
Mean (SD^2^)	9.4 (5.1)	8.9 (4.5)	11.3 (7.7)	9.3 (5.0)
Median	8.6	8.2	9.1	8.6
Min - Max	0.0–17.9	3.0–22.2	4.7–22.5	0.0–22.5
**Sow slaughter rate**,** %**				
Mean (SD^2^)	50.6 (42.3)	33.3 (14.7)	32.6 (23.9)	41.5 (32.3)
Median	38.2	31.4	39,6	36.0
Min - Max	20.5–160.0	4.4–64.7	0.0–51.3	0.0–160.0
**Sikava Welfare index scores**				
Mean (SD^2^)	86.7 (10.0)	89.4 (10.2)	93.2 (8.3)	88.6 (10.0)
Median	85.2	93.7	89.2	87.7
Min - Max	70.2–102.0	71.3–103.5	87.7–102.8	70.2–103.5
**External biosecurity** ^**3**^				
Mean (SD^2^)	77 (6)	76 (11)	80 (14)	77 (9)
Median	77	76	85	78
Min - Max	67–89	55–96	60–91	55–96
**Internal biosecurity** ^**3**^				
Mean (SD^2^)	57 (11)	57 (11)	71 (8)	58 (11)
Median	58	58	73	58
Min - Max	37–79	40–75	61–78	37–79
**Total biosecurity** ^**3**^				
Mean (SD^2^)	67 (8)	66 (10)	76 (11)	68 (9)
Median	67	64	80	67
Min - Max	57–83	51–85	61–84	51–85
**Free lactation** ^**4**^				
Yes	9	11	1	21
No	13	9	3	25
**Environment index** ^**5**^				
Mean (SD^2^)	6 (1.3)	6 (1.6)	5 (0.5)	6 (1.4)
Median	6	6	5	6
Min - Max	5–9	5–10	5–6	5–10
**Enrichment index** ^**6**^				
Mean (SD^2^)	6 (1.3)	6 (1.7)	6 (0.8)	6 (1.4)
Median	6	6	6	6
Min - Max	4–9	3–9	5–7	3–9
**Sows with minor + severe shoulder lesions**,** %**^**7**^				
Mean (SD^2^)	14 (13.9)	14 (14.1)	5 (4.5)	13 (13.5)
Median	7	7	5	7
Min - Max	2–53	0–46	0–10	0–53
**Pregnant sows with condition score 1–2**,** %**				
Mean (SD^2^)	3 (4.6)	3 (3.4)	0 (0.0)	3 (3.9)
Median	2	1	0	0
Min - Max	0–17	0–12	0–0	0–17
**Lactating sows with condition score 1–2**,** %**				
Mean (SD^2^)	9 (11.9)	7 (8.1)	5 (10.0)	8 (10.1)
Median	4	4	0	4
Min - Max	0–40	0–26	0–20	0–40

^1^ McGlone (2006) [25]

^2^ Standard deviation

^3^ Biosecurity scores based on risk-based tool Biocheck.UGent™ (www.biocheck.ugent.be). The veterinarian had not evaluated the biosecurity in two herds during the study year

^4^ Free lactation included both temporary crating and free farrowing systems. The register data did not include information for two herds

^5^ The environment index (min = 5, max = 15) summed the poorest scores for air quality, pen cleanliness, operational condition of housing structures, water and feeding equipment, and animal density

^6^ The enrichment index (min = 3, max = 9) included the poorest scores for the amount of bedding and enrichment for lactating and pregnant sows and nesting material for farrowing sows

^7^ The veterinarian inspected 30 newly weaned sows (or the entire group if the group size was smaller)

### AMU

**Fig. 1:**
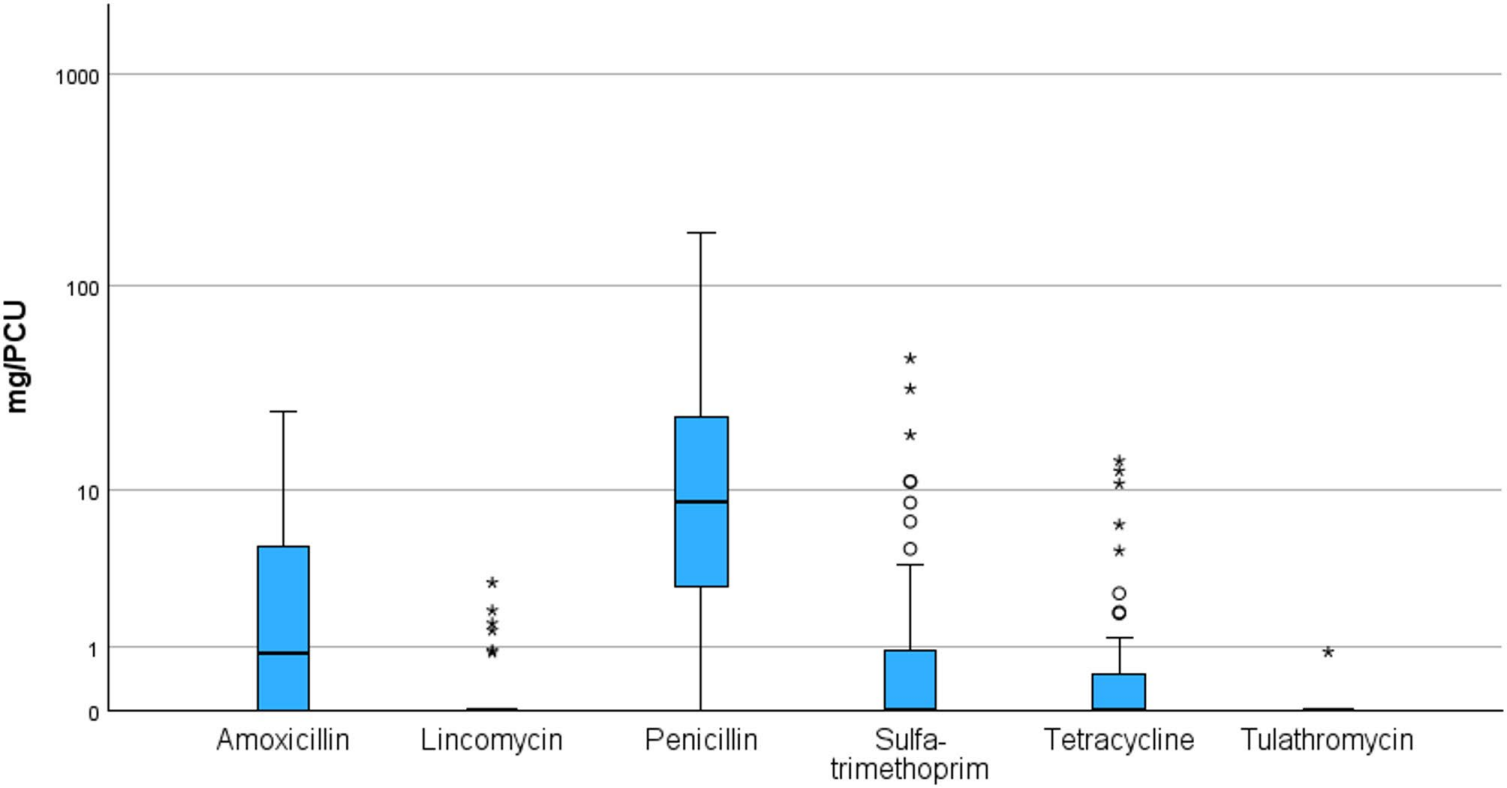
AMU for sows in 48 herds expressed as mg/PCU by different active substances (log-transformed scale)

All medications were administered to sows individually as injections except tetracycline spray treatments topically for skin indications. The median total AMU for the sows in the study herds was 21.9 mg/PCU (range 0.3-178.5). Penicillin was used in 92% of the herds and was consistently the most administered antimicrobial in the study herds. Amoxicillin, sulfa-trimethoprim, tetracycline, lincomycin, and tulathromycin were used in 52%, 35%, 31%, 13%, and 2% of the herds, respectively (Figs. 1).

### Treatment indications and antimicrobials used

The sows were mainly treated for locomotory (34%) and udder (20%) disorders using injectable penicillin, the most commonly used antimicrobial in the study herds (50%) (Table 3). Three herds were excluded from the indication data as they had not recorded treatments individually.

**Table 3:** Descriptive data of treatment indication groups and antimicrobials used for sows during 2022 (45 herds)

		*N* (%) of treatment courses treated with					
Indication group	*N* (%) of treatment courses	PEN	AMO	TET	STR	LIN	TUL
Locomotory	3022 (34)	2124 (70)	542 (18)	246 (8)	3 (0)	107(4)	0 (0)
Udder	1780 (20)	1149 (65)	375 (21)	43 (2)	213 (12)	0 (0)	0 (0)
Reproductive	1105 (12)	261 (24)	642 (59)	11 (1)	191 (17)	0 (0)	0 (0)
Skin	994 (11)	154 (16)	25 (2)	815 (82)	0 (0)	0 (0)	0 (0)
General infection	128 (1)	17 (13)	6 (5)	0 (0)	105 (82)	0 (0)	0 (0)
Digestive	120 (1)	76 (64)	6 (5)	26 (22)	8 (7)	0 (0)	4 (3)
Respiratory	130 (2)	15 (12)	3 (2)	1 (1)	0 (0)	0 (0)	111 (85)
Fever	420 (5)	168 (40)	217 (52)	0 (0)	35 (8)	0 (0)	0 (0)
Loss of appetite	716 (8)	112 (16)	306 (43)	2 (0)	294 (41)	0 (0)	2 (0)
Other	505 (6)	385 (76)	119 (24)	0 (0)	0 (0)	0 (0)	1 (0)
Total	8920 (100)	4461 (50)	2241 (25)	1144 (13)	849 (10)	107(1)	118 (1)

*PEN: Penicillin, AMO: Amoxicillin, TET: Tetracycline, STR: Sulfa-trimethoprim, LIN: Lincomycin, TUL: Tulathromycin

### Statistical analyses

#### Univariate analyses

Correlations among all predictor variables were tested using the Spearman correlation test. Total AMU in sows, expressed as mg/PCU, was significantly correlated with herd size, sow slaughter rate, and biosecurity scores (Table 4). All correlations are presented in Table 4.

**Table 4:** Summarized Spearman correlations between predictor variables in the study investigating antimicrobial use in Finnish sows.

	AMU	FL	HS	SM	SR	BE	BI	BT	SWI	EI	ERI	NM	SL	BC1	BC2
**AMU**															
**FL**	**.13**														
**HS**	**.36** ^**2**^	.06													
**SM**	**.20**	.15	**.20**												
**SR**	**.36** ^**2**^	**.16**	**.21**	.06											
**BE**	**.40** ^**1**^	**.34** ^**2**^	**.25**	**.09**	**.11**										
**BI**	**.58** ^**1**^	**.10**	**.49** ^**1**^	**.27**	**.38** ^**1**^	**.59** ^**1**^									
**BT**	**.52** ^**1**^	**.22**	**.43** ^**1**^	**.20**	**.31** ^**2**^	**.86** ^**1**^	**.89** ^**1**^								
**SWI**	**.03**	**.60** ^**1**^	.17	.43^1^	**.11**	**.42** ^**1**^	**.12**	**.26**							
**EI**	.01	.11	**.12**	.24	.10	.06	.18	.14	.26						
**ERI**	.12	.16	**.23**	.14	**.02**	**.01**	.02	**.04**	.24	.22					
**NM**	.28	.26	**.05**	.17	.25	.05	.06	.02	.23	**.14**	**.79** ^**1**^				
**SL**	.13	**.05**	.13	.25	**.13**	.30^2^	.13	.20	.42^1^	**.33** ^**2**^	**.19**	**.11**			
**BC1**	.10	.07	**.05**	**.26**	.05	.05	.14	.08	.42^1^	**.39** ^**1**^	**.09**	.09	**.15**		
**BC2**	.14	.03	.19	**.10**	.23	.12	.26	.22	.38^1^	**.32** ^**2**^	.00	.02	**.25** ^**2**^	**.58** ^**1**^	

AMU: total AMU in sows expressed as milligrams of antimicrobials administered per population corrected unit (mg/PCU)

FL: score describing prevalence of free lactation, included both temporary crating and free farrowing systems.

HS: herd side

SM: sows’ mortality (%) during the study period

SR: slaughter rate (%) during the study period

BE: external biosecurity score

BI: internal biosecurity score

BT: total biosecurity score

SWI: score describing the welfare of sows; higher scores indicate better welfare

EI: environment index sums the highest scores from the veterinarians’ assessments of air quality, pen cleanliness, operational condition of structures, water and feeding equipment, and animal density; higher scores indicate poorer assessments

ERI: enrichment index sums the highest scores from the veterinarians’ assessments of the amount of bedding and enrichment for lactating and pregnant sows and nesting material for farrowing sows

NM: the highest scores from the veterinarians’ assessments of amount of nesting material for farrowing sows

SL: % of sows with minor + severe shoulder lesions

BC1: body condition score 1 and 2 for lactating sows (%)

BC2: body condition score 1 and 2 for pregnant sows (%)

Bolded values indicate positive correlations

^1^ Correlation is significant at the 0.01 level

^2^ Correlation is significant at the 0.05 level

#### Multivariate analyses

Model 1: Total AMU in sows expressed as mg/PCU was predicted by herd size (*p* < 0.001) and herd type (*p* = 0.04). The AMU was higher in large herds (predicted mg/PCU 44.3 ± SE 7.7) compared with small (20.7 ± 8.3; *p* = 0.02) and medium-sized herds (8.0 ± 7.7; *p* < 0.001). Piglet-producing herds (*n* = 23) tended to have a higher AMU (predicted mg/PCU 39.4 ± 6.0; *p* = 0.05) than both farrow-to-finish farms (*n* = 21) (24.3 ± 6.6; *p* = 0.05) and sow pools (*n* = 4) (9.3 ± 14.7; *p* = 0.05) in pair-wise comparisons.

Model 2: Total AMU in sows expressed as mg/PCU increased with increasing internal biosecurity scores (1.32% per point; *p* < 0.001). Herd type was also significant (*p* = 0.03), with predicted higher values for piglet-producing herds (39.2 ± SE 6.3) than farrow-to-finish herds (23.6 ± 6.8, *p* = 0.04) and a similar tendency between piglet-producing herds and sow pools (8.6 ± 15.4, *p* = 0.06).

## Discussion

Our study revealed that the amount of AMU in sows varied between herds and was lower than that reported in the previous Finnish study by Sali et al. [12]. The selection of active ingredients for different indications aligned with national recommendations. Most treatments were administered for locomotory, udder, and reproductive disorders. AMU was associated with herd size, type, and biosecurity.

We found a large variation in AMU in sows between herds (Fig. 1). This was also observed in previous Finnish studies by Sali et al. [12] and Yun et al. [13] and in studies from other countries such as Sweden, Germany, and the Netherlands [8–10]. Such variation may be due to differences in management practices and herd-specific factors, such as levels of hygiene, prophylactic AMU, and treatment decisions made by farmers or veterinarians [33].

According to treatment records kept by the farm, all antimicrobial treatments were administered only parenterally to individual sows. International comparisons of the overall sales of veterinary antimicrobial agents to all food-producing animal species have shown that oral solutions were the highest-selling product form [7]. However, group treatments are usually given to young growing animals, not adults [11, 34, 35]. In addition, Finland has had a strict approach to group medication dating back to 1996, when the first recommendations for antimicrobial use for animals were provided [36].

In this study, penicillin was the most administered antimicrobial, followed by amoxicillin and sulfa-trimethoprim, showing that the selection of antimicrobials for treatment followed national [37] and European [38] prudent AMU guidelines. Previous studies from Denmark, Sweden, and Finland revealed that penicillin is a commonly used antimicrobial in sows [10, 12, 13, 16], while reports from Germany [9, 39] revealed that sows were mostly treated with tetracyclines. In the present study, use of tetracyclines was rather low, consistent with a previous study by Sali et al. [12]. The use of amoxicillin and sulfa-trimethoprim was also consistent with previous Finnish studies [12, 13]. In this study, fluoroquinolones and third- and fourth-generation cephalosporins were not used at all, as expected, because their use is restricted by Finnish national legislation and can only be justified if there is no other effective treatment [28]. Sikava has also banned use of these antimicrobials in 2021 [14]. Fluoroquinolones and third- and fourth-generation cephalosporins are also restricted by law in other Nordic countries, such as Sweden [11]. Fluoroquinolones are also restricted in Denmark [16]. In contrast, Irish researchers found that injectable fluoroquinolones were used in 83.6% of pig herds [35], although they did not report the use separately for sows. The use of tulathromycin and lincomycin was also very low in the present study. An earlier Finnish study by Yun et al. [13] reported slightly higher use of macrolides and lincosamides and some use of fluoroquinolones. Lincosamide use was also low in other European countries, such as Belgium, France, Germany, and Sweden [11]. In contrast, use of macrolides and fluoroquinolones was clearly higher; macrolides were used in 4.5% and fluoroquinolones for 9.0% of treatments in breeding pigs [11].

Antimicrobials were administered mainly to treat locomotory, udder, reproductive, and skin-related disorders. Diagnosing the cause of lameness in sows is not easy, partly because their large joints are covered with muscle, and it is difficult to use diagnostic methods such as radiography in swine practice [40]. Treatment decision is typically based on visual inspection, palpation, and rectal temperature measurement [40], which can lead to unnecessary AMU for locomotory disorders. Postpartum dysgalactia syndrome (PDS) is the most commonly used term for udder and reproductive-tract disorders around farrowing [41]. The primary treatment recommendation for PDS is non-steroidal anti-inflammatory drugs (NSAIDs) with oxytocin to support milk production; antimicrobials should only be used if NSAIDs and oxytocin treatment are unsuccessful, or the signs are severe [41]. However, a Swedish study revealed that PDS is still the main reason for antimicrobial treatment for sows [42]. In our study, tetracycline spray was very commonly used to treat skin infections (82% of treatments, including shoulder lesions). Kaiser et al. reported that topical zinc ointment with rubber mats significantly reduced the size of shoulder ulcers on days 14 and 21, compared to both day one and treatment with the antimicrobial spray [43].

In this study, the owners treated sows with antimicrobials for poor appetite or fever. Poor appetite can be associated with almost any disease or condition in the sow [40, 44, 45], which raises the question of whether it is appropriate to use antimicrobials in a sow that has not eaten and has no other signs. Poor appetite in pigs may be due to stomach ulcers, stress, or poor-quality feed [46], for which antimicrobials are not effective. Both poor appetite and fever require detailed clinical examination and more accurate diagnosis. AMU was low for other indications in this study. For example, only one herd had treated sows with antimicrobials due to respiratory disorders, as expected, as the prevalence of infectious swine diseases in Finland is generally low [47].

We sought to identify farm-level risk factors associated with AMU and showed that increasing herd size was significantly associated with increasing AMU, similar to other studies [48, 49]. However, some studies did not reveal associations between herd size and AMU [5, 12, 50]. A comparison would have been easier if the studies used a similar definition of herd size, method of AMU quantification, or both. A study by van der Fels-Klerx et al. showed that a larger number of pigs present on the farm may result in a higher probability of infection, which may explain the increased AMU on large farms [51]. Previous studies have reported a higher prevalence of infection on larger farms [52, 53].

AMU for sows calculated as mg/PCU was higher in piglet-producing herds than in farrow-to-finish herds. Our results contrast with a German study that reported the factor “farm category” as having no effect on AMU in sows [48]. However, it is challenging to compare the results with studies from other countries due to different definitions of herd types and different ways of calculating AMU [34, 48, 51]. We cannot explain any biologically plausible reason for this finding. Even though our data included many possible risk factors, we may have missed important ones to explain this result. We did not have any information, for example, about origin of gilts, breed, feeding, or sow ages.

According to our results, AMU expressed as mg/PCU increased with increasing internal biosecurity scores. A study by Raasch et al. reported similar results, with herds with higher TI in breeding pigs scoring higher on internal biosecurity levels [54]. A possible explanation for this association could be increased internal biosecurity measures due to the current high infection pressure in the herds, resulting in higher AMU [54]. They also speculated that farmers owning a farm with a higher internal biosecurity score may have a more cautious approach and may have a lower threshold to use antimicrobial treatment [54]. The positive association between AMU and biosecurity is inconsistent with studies from other countries, as previous studies have shown a decrease in AMU as biosecurity improves [20–22]. A study by Laanen et al. was based on the Biocheck questionnaire and revealed that improvements in internal biosecurity were highly likely to have an effect on the reduction of prophylactic AMU [21]. Previous studies have shown that farms were able to control the impact of infectious diseases using other ways than antimicrobials, such as vaccinations and better biosecurity [55, 56]. Mallioris et al. found that factors related to internal biosecurity had the largest impact on AMU in pigs compared with on-farm practices and characteristics, such as external biosecurity, swine husbandry, and vaccination [57].

Surprisingly few farm-level factors remained in the model, although a large number (14 factors) were tested. It is likely that many of these factors were excluded from the model because they correlated with those that remained in the model (Table 4). It is also possible that our data collection measures were not sufficiently accurate, as the methods for measuring the various factors are rather imprecise due to the coarse scale of the assessment and the large number of assessors. In addition, the sample size may have been too small because we could not estimate in advance how much of an impact these factors might have, and the sample size was not calculated accordingly. However, previous studies revealed that improvements in pig welfare and housing conditions can reduce AMU in pigs [19, 50].

Inclusion of the veterinarian as a random factor improved the fit of our models, indicating that veterinarian may be associated with AMU. In a German study by Van Rennings et al., the veterinarian alone had a significant effect on treatment frequency, and farm size had a significant effect on treatment frequency only in interaction with the veterinarian [9]. In Germany, where many veterinarians work with pigs, some specialize in fattening pigs and others in sows and piglets, resulting in different treatment strategies [9]. In addition, veterinarians advise farms of different sizes with different management strategies [9]. Previous studies have shown that the attitudes and practices of the veterinarians themselves influence AMU on farms [58, 59]. The relationship and communication between the veterinarian and the farmer are also important factors in relation to AMU on farms [23, 58].

Our study had some limitations, especially due to the challenges associated with using the register data. These include data accuracy, the diversity of assessments, and the different ways and practices of recording data in the herd health register by veterinarians and farmers. These limitations were considered when processing, reporting, and discussing the data.

## Conclusion

We observed that AMU (mg/PCU) in sows varied widely between herds but was moderate compared with results from other European countries. Sows were mostly treated individually for locomotory, udder, and reproductive disorders. The most common antimicrobial used in sows was injectable penicillin. Potential farm-level risk factors that may be associated with AMU include herd size, herd type, and internal biosecurity scores. However, further research into farm-level risk factors is needed to find ways to improve sow health in herds and thus reduce the need for antimicrobials.

## Data Availability

No datasets were generated or analysed during the current study.
